# Diaqua­bis[5-(2-pyrid­yl)-1*H*-tetra­zolato-κ^2^ *N* ^1^,*N* ^5^]cobalt(II)

**DOI:** 10.1107/S160053680900470X

**Published:** 2009-02-18

**Authors:** Zhen-Hai Sun, Ling-Bo Meng, Hua Lin

**Affiliations:** a School of Chemistry and Life Sciences, Harbin University, Harbin 150080, People’s Republic of China

## Abstract

In the title compound, [Co(C_6_H_4_N_5_)_2_(H_2_O)_2_], the Co atom is bonded to two water mol­ecules and two bidentate 5-(2-pyrid­yl)tetra­zolate ligands resulting in a slightly distorted octa­hedral CoN_4_O_2_ coordination geometry. The Co^II^ cation is situated on a crystallographic center of inversion. The asymmetric unit therefore comprises one-half of the mol­ecule. The four N atoms belonging to two bidentate 5-(2-pyrid­yl)tetra­zolate ligands lie in the equatorial plane and the two associated water mol­ecules are observed in the axial coordination sites. The crystal structure exhibits a three-dimensional supra­molecular network assembled by inter­molecular O—H⋯N hydrogen bonds.

## Related literature

For general background, see: Caneschi *et al.* (1989 ▶); Tsukuda *et al.* (2002 ▶); Vostrikova *et al.* (2000 ▶); Kuchar *et al.* (2003 ▶)
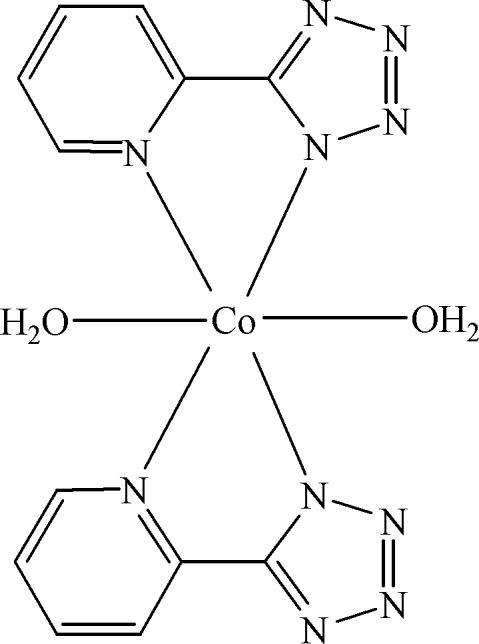


## Experimental

### 

#### Crystal data

[Co(C_6_H_4_N_5_)_2_(H_2_O)_2_]
*M* *_r_* = 387.25
Monoclinic, 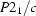

*a* = 7.999 (2) Å
*b* = 12.870 (3) Å
*c* = 7.168 (2) Å
β = 95.99 (1)°
*V* = 733.8 (3) Å^3^
*Z* = 2
Mo *K*α radiation
μ = 1.20 mm^−1^
*T* = 296 K
0.12 × 0.10 × 0.08 mm

#### Data collection

Bruker APEXII CCD area-detector diffractometer
Absorption correction: multi-scan (*SADABS*; Bruker, 2001 ▶) *T*_min_ = 0.869, *T*_max_ = 0.910
3854 measured reflections
1346 independent reflections
1270 reflections with *I* > 2σ(*I*)
*R*_int_ = 0.012

#### Refinement

*R*[*F* ^2^ > 2σ(*F* ^2^)] = 0.026
*wR*(*F* ^2^) = 0.073
*S* = 1.00
1346 reflections
122 parameters
3 restraints
H atoms treated by a mixture of independent and constrained refinement
Δρ_max_ = 0.29 e Å^−3^
Δρ_min_ = −0.39 e Å^−3^

### 

Data collection: *APEX2* (Bruker, 2004 ▶); cell refinement: *SAINT-Plus* (Bruker, 2001 ▶); data reduction: *SAINT-Plus*; program(s) used to solve structure: *SHELXS97* (Sheldrick, 2008 ▶); program(s) used to refine structure: *SHELXL97* (Sheldrick, 2008 ▶); molecular graphics: *SHELXTL* (Sheldrick, 2008 ▶); software used to prepare material for publication: *SHELXTL*.

## Supplementary Material

Crystal structure: contains datablocks I, global. DOI: 10.1107/S160053680900470X/im2097sup1.cif

Structure factors: contains datablocks I. DOI: 10.1107/S160053680900470X/im2097Isup2.hkl

Additional supplementary materials:  crystallographic information; 3D view; checkCIF report

## Figures and Tables

**Table 1:** Hydrogen-bond geometry (Å, °)

*D*—H⋯*A*	*D*—H	H⋯*A*	*D*⋯*A*	*D*—H⋯*A*
O1*W*—H2*W*⋯N2^i^	0.82 (1)	2.00 (1)	2.798 (2)	168 (4)
O1*W*—H1*W*⋯N1^ii^	0.82 (1)	1.92 (1)	2.736 (2)	179 (3)

Symmetry codes: (i) 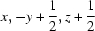; (ii) 
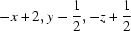
.

**Fig. 1.**
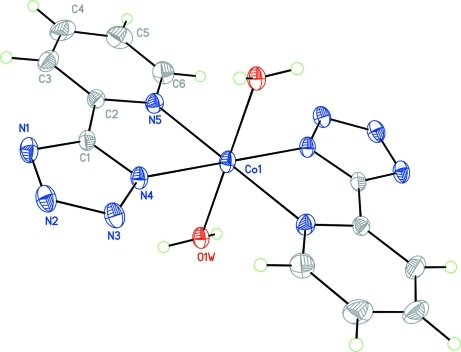
The molecular structure of (I), around CoII, displacement ellipsoids for the non-hydrogen atoms are drawn at the 50% probability level.

**Fig. 2.**
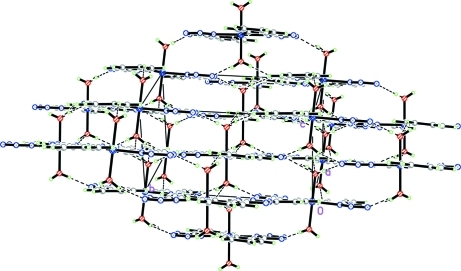
Packing diagram of (I) showing the hydrogen bond interaction.

## supplementary crystallographic information

### Comment

The design of different kinds of paramagnetic metal coordination architectures with appropriate organic radicals and coligands has been an important subject during the last decade because of its potential usages for molecule-based magnetic materials and optical devices (Caneschi *et al.*, 1989; Tsukuda *et al.*, 2002; Vostrikova *et al.*, 2000; Kuchar *et al.*, 2003). If organic radicals such as the tridentate nitronyl nitroxide radical or the bidentate nitroxide radical are used as an integral part of a ligand system a large number of building blocks with various potentional applications may be achieved. In this paper, we report the structure of the title compound, (I).

The molecular structure of the title compound is shown in Fig. 1. The Co^II^ atom (site symmetry 1) is bonded to two water molecules and two bidentate 5-(2-pyridyl)tetrazolato ligands resulting in a slightly distorterd octahedral CoN_4_O_2_ coordination geometry. The Co^II^ cation is situated on a crystallographic center of inversion. The asymmetric unit therefore comprises one half of the molecule. The four nitrogen atoms belonging to two bidentate 5-(2-pyridyl)tetrazolato ligands lie in the equatorial plane and the two associated water molecules are observed in the axial coordination sites. In the equatorial plane, the Co—N bond lengths are in the range of 2.142 (2)–2.173 (2) Å. The Co—O axial bond length is 2.093 (2) Å. It is also worth noticing that the three-dimensional supramolecular structure is assembled *via* complicated hydrogen bonds, shown in Fig. 2. The hydrogen bonds are listed in Table 1.

### Experimental

A mixture of cobalt(II) dichloride hexhydrate (1 mmoL), 5-(2-pyridyl)tetrazolate (1 mmoL) in 20 ml mixed solvate(1:1) of methanol and water was refluxed for several hours. After cooling down the solution was filterated and the filtrate was kept in the ice box. One week later, red blocks of (I) were obtained with a yield of *ca* 56%. Anal. Calc. for C_12_H_12_CoN_10_O_2_: C 37.19, H 3.10, N 36.15%; Found: C 37.22, H 3.08, N 36.11%.

### Refinement

All H atoms were placed in calculated positions with C—H = 0.93Å and refined as riding with *U*_iso_(H) = 1.2*U*_eq_(C). The H atoms of the water molecule were located from difference density maps and were refined with distance restraints of d(H–H) = 1.38 (2) Å, d(O–H) = 0.82 (1) Å.

### Crystal data

**Table:**

[Co(C_6_H_4_N_5_)_2_(H_2_O)_2_]	*F*(000) = 394
*M**_r_* = 387.25	*D*_x_ = 1.752 Mg m^−^^3^
Monoclinic, *P*2_1_/*c*	Mo *K*α radiation, λ = 0.71073 Å
Hall symbol: -P 2ybc	Cell parameters from 1346 reflections
*a* = 7.999 (2) Å	θ = 2.6–25.5°
*b* = 12.870 (3) Å	µ = 1.20 mm^−^^1^
*c* = 7.168 (2) Å	*T* = 296 K
β = 95.99 (1)°	Block, red
*V* = 733.8 (3) Å^3^	0.12 × 0.10 × 0.08 mm
*Z* = 2	

### Data collection

**Table:**

Bruker APEXII CCD area-detector diffractometer	1346 independent reflections
Radiation source: fine-focus sealed tube	1270 reflections with *I* > 2σ(*I*)
graphite	*R*_int_ = 0.012
φ and ω scans	θ_max_ = 25.5°, θ_min_ = 2.6°
Absorption correction: multi-scan (*SADABS*; Bruker, 2001)	*h* = −9→7
*T*_min_ = 0.869, *T*_max_ = 0.910	*k* = −15→12
3854 measured reflections	*l* = −8→7

### Refinement

**Table:**

Refinement on *F*^2^	Secondary atom site location: difference Fourier map
Least-squares matrix: full	Hydrogen site location: inferred from neighbouring sites
*R*[*F*^2^ > 2σ(*F*^2^)] = 0.026	H atoms treated by a mixture of independent and constrained refinement
*wR*(*F*^2^) = 0.073	*w* = 1/[σ^2^(*F*_o_^2^) + (0.036*P*)^2^ + 0.7827*P*] where *P* = (*F*_o_^2^ + 2*F*_c_^2^)/3
*S* = 1.00	(Δ/σ)_max_ < 0.001
1346 reflections	Δρ_max_ = 0.29 e Å^−^^3^
122 parameters	Δρ_min_ = −0.38 e Å^−^^3^
3 restraints	Extinction correction: *SHELXL97* (Sheldrick, 2008), Fc^*^=kFc[1+0.001xFc^2^λ^3^/sin(2θ)]^-1/4^
Primary atom site location: structure-invariant direct methods	Extinction coefficient: 0.032 (2)

### Special details

**Table:**

Geometry. All e.s.d.'s (except the e.s.d. in the dihedral angle between two l.s. planes) are estimated using the full covariance matrix. The cell e.s.d.'s are taken into account individually in the estimation of e.s.d.'s in distances, angles and torsion angles; correlations between e.s.d.'s in cell parameters are only used when they are defined by crystal symmetry. An approximate (isotropic) treatment of cell e.s.d.'s is used for estimating e.s.d.'s involving l.s. planes.
Refinement. Refinement of *F*^2^ against ALL reflections. The weighted *R*-factor *wR* and goodness of fit *S* are based on *F*^2^, conventional *R*-factors *R* are based on *F*, with *F* set to zero for negative *F*^2^. The threshold expression of *F*^2^ > σ(*F*^2^) is used only for calculating *R*-factors(gt) *etc*. and is not relevant to the choice of reflections for refinement. *R*-factors based on *F*^2^ are statistically about twice as large as those based on *F*, and *R*- factors based on ALL data will be even larger.

### Fractional atomic coordinates and isotropic or equivalent isotropic displacement parameters (Å^2^)

**Table:**

	*x*	*y*	*z*	*U*_iso_*/*U*_eq_	
Co1	1.0000	0.0000	0.0000	0.02558 (17)	
C1	0.9245 (2)	0.22105 (14)	0.0495 (2)	0.0238 (4)	
C2	0.7797 (2)	0.17179 (15)	0.1175 (2)	0.0252 (4)	
C3	0.6476 (3)	0.22658 (19)	0.1749 (3)	0.0362 (5)	
H3	0.6448	0.2988	0.1690	0.043*	
C4	0.5198 (3)	0.1717 (2)	0.2411 (3)	0.0459 (6)	
H7	0.4276	0.2062	0.2805	0.055*	
C5	0.5285 (3)	0.0653 (2)	0.2492 (4)	0.0473 (6)	
H6	0.4434	0.0273	0.2963	0.057*	
C6	0.6639 (3)	0.01547 (19)	0.1872 (3)	0.0377 (5)	
H5	0.6690	−0.0567	0.1921	0.045*	
N1	0.9563 (2)	0.32120 (13)	0.0376 (2)	0.0303 (4)	
N2	1.1049 (2)	0.32543 (13)	−0.0308 (2)	0.0316 (4)	
N3	1.1587 (2)	0.23204 (13)	−0.0591 (2)	0.0290 (4)	
N4	1.0465 (2)	0.16372 (12)	−0.0086 (2)	0.0246 (4)	
N5	0.7867 (2)	0.06737 (13)	0.1208 (2)	0.0270 (4)	
O1W	1.13421 (19)	−0.00907 (10)	0.2663 (2)	0.0275 (3)	
H1W	1.108 (5)	−0.0604 (13)	0.324 (4)	0.080*	
H2W	1.141 (5)	0.0450 (12)	0.326 (4)	0.080*	

### Atomic displacement parameters (Å^2^)

**Table:**

	*U*^11^	*U*^22^	*U*^33^	*U*^12^	*U*^13^	*U*^23^
Co1	0.0296 (2)	0.0178 (2)	0.0309 (2)	−0.00067 (13)	0.01065 (16)	−0.00120 (13)
C1	0.0315 (10)	0.0199 (9)	0.0195 (8)	0.0036 (7)	0.0005 (7)	−0.0013 (7)
C2	0.0291 (10)	0.0273 (10)	0.0190 (9)	0.0042 (8)	0.0011 (7)	−0.0026 (7)
C3	0.0361 (12)	0.0399 (12)	0.0325 (11)	0.0140 (9)	0.0033 (9)	−0.0038 (9)
C4	0.0289 (11)	0.0691 (18)	0.0408 (13)	0.0128 (11)	0.0090 (9)	−0.0086 (12)
C5	0.0300 (12)	0.0680 (18)	0.0465 (13)	−0.0078 (11)	0.0160 (10)	−0.0051 (12)
C6	0.0344 (12)	0.0375 (12)	0.0431 (13)	−0.0079 (9)	0.0125 (10)	−0.0014 (9)
N1	0.0448 (10)	0.0194 (8)	0.0263 (9)	0.0029 (7)	0.0020 (7)	0.0005 (7)
N2	0.0447 (10)	0.0207 (8)	0.0295 (9)	−0.0039 (7)	0.0038 (7)	0.0018 (7)
N3	0.0371 (9)	0.0218 (8)	0.0289 (8)	−0.0062 (7)	0.0070 (7)	0.0005 (7)
N4	0.0304 (8)	0.0177 (8)	0.0267 (8)	−0.0016 (7)	0.0081 (6)	−0.0003 (6)
N5	0.0271 (8)	0.0272 (9)	0.0278 (8)	0.0000 (7)	0.0084 (7)	−0.0021 (6)
O1W	0.0340 (8)	0.0203 (7)	0.0289 (7)	−0.0014 (6)	0.0073 (6)	−0.0008 (5)

### Geometric parameters (Å, °)

**Table:**

Co1—O1W^i^	2.0932 (16)	C3—H3	0.9300
Co1—O1W	2.0932 (16)	C4—C5	1.372 (4)
Co1—N4^i^	2.1416 (16)	C4—H7	0.9300
Co1—N4	2.1416 (16)	C5—C6	1.371 (3)
Co1—N5^i^	2.1726 (16)	C5—H6	0.9300
Co1—N5	2.1726 (16)	C6—N5	1.317 (3)
C1—N1	1.318 (3)	C6—H5	0.9300
C1—N4	1.325 (3)	N1—N2	1.333 (3)
C1—C2	1.448 (3)	N2—N3	1.300 (3)
C2—N5	1.345 (3)	N3—N4	1.333 (2)
C2—C3	1.369 (3)	O1W—H1W	0.817 (10)
C3—C4	1.368 (4)	O1W—H2W	0.815 (10)
			
O1W^i^—Co1—O1W	180.00 (8)	C2—C3—H3	121.1
O1W^i^—Co1—N4^i^	90.41 (6)	C3—C4—C5	119.6 (2)
O1W—Co1—N4^i^	89.59 (6)	C3—C4—H7	120.2
O1W^i^—Co1—N4	89.59 (6)	C5—C4—H7	120.2
O1W—Co1—N4	90.41 (6)	C6—C5—C4	119.4 (2)
N4^i^—Co1—N4	180.000 (15)	C6—C5—H6	120.3
O1W^i^—Co1—N5^i^	90.47 (6)	C4—C5—H6	120.3
O1W—Co1—N5^i^	89.53 (6)	N5—C6—C5	121.6 (2)
N4^i^—Co1—N5^i^	76.39 (6)	N5—C6—H5	119.2
N4—Co1—N5^i^	103.61 (6)	C5—C6—H5	119.2
O1W^i^—Co1—N5	89.53 (6)	C1—N1—N2	104.43 (16)
O1W—Co1—N5	90.47 (6)	N3—N2—N1	110.03 (15)
N4^i^—Co1—N5	103.61 (6)	N2—N3—N4	108.90 (16)
N4—Co1—N5	76.39 (6)	C1—N4—N3	104.89 (16)
N5^i^—Co1—N5	180.00 (11)	C1—N4—Co1	113.77 (13)
N1—C1—N4	111.76 (18)	N3—N4—Co1	141.31 (13)
N1—C1—C2	128.05 (18)	C6—N5—C2	118.81 (18)
N4—C1—C2	120.19 (18)	C6—N5—Co1	126.01 (15)
N5—C2—C3	122.8 (2)	C2—N5—Co1	115.11 (13)
N5—C2—C1	114.22 (17)	Co1—O1W—H1W	112 (2)
C3—C2—C1	123.0 (2)	Co1—O1W—H2W	115 (2)
C4—C3—C2	117.8 (2)	H1W—O1W—H2W	115.5 (19)
C4—C3—H3	121.1		

Symmetry codes: (i) −*x*+2, −*y*, −*z*.

### Hydrogen-bond geometry (Å, °)

**Table:**

*D*—H···*A*	*D*—H	H···*A*	*D*···*A*	*D*—H···*A*
O1W—H2W···N2^ii^	0.82 (1)	2.00 (1)	2.798 (2)	168 (4)
O1W—H1W···N1^iii^	0.82 (1)	1.92 (1)	2.736 (2)	179 (3)

Symmetry codes: (ii) *x*, −*y*+1/2, *z*+1/2; (iii) −*x*+2, *y*−1/2, −*z*+1/2.
